# Visualization by High Resolution Immunoelectron Microscopy of the Transient Receptor Potential Vanilloid-1 at Inhibitory Synapses of the Mouse Dentate Gyrus

**DOI:** 10.1371/journal.pone.0119401

**Published:** 2015-03-16

**Authors:** Miren-Josune Canduela, Juan Mendizabal-Zubiaga, Nagore Puente, Leire Reguero, Izaskun Elezgarai, Almudena Ramos-Uriarte, Inmaculada Gerrikagoitia, Pedro Grandes

**Affiliations:** 1 Department of Neurosciences, Faculty of Medicine and Dentistry, University of the Basque Country UPV/EHU, E-48940, Leioa, Spain; 2 Achucarro Basque Center for Neuroscience, Bizkaia Science and Technology Park, Zamudio, Spain; St. Joseph's Hospital and Medical Center, UNITED STATES

## Abstract

We have recently shown that the transient receptor potential vanilloid type 1 (TRPV1), a non-selective cation channel in the peripheral and central nervous system, is localized at postsynaptic sites of the excitatory perforant path synapses in the hippocampal dentate molecular layer (ML). In the present work, we have studied the distribution of TRPV1 at inhibitory synapses in the ML. With this aim, a preembedding immunogold method for high resolution electron microscopy was applied to mouse hippocampus. About 30% of the inhibitory synapses in the ML are TRPV1 immunopositive, which is mostly localized perisynaptically (∼60% of total immunoparticles) at postsynaptic dendritic membranes receiving symmetric synapses in the inner 1/3 of the layer. This TRPV1 pattern distribution is not observed in the ML of TRPV1 knock-out mice. These findings extend the knowledge of the subcellular localization of TRPV1 to inhibitory synapses of the dentate molecular layer where the channel, in addition to excitatory synapses, is present.

## Introduction

TRPV1 plays an important role in peripheral pain perception and modulates neurotransmitter release and synaptic plasticity in the brain. It is a ligand-gated nonselective cationic channel activated by high temperature, low pH, some natural products (resiniferatoxin, capsaicin) and endogenous lipids or endovanilloids [1–5]. The structure of TRPV1 is now revealed [6,7] and its presence in many regions of the central nervous system (CNS) has been reported over the years [8–14] where it participates in physiological functions such as synaptic plasticity [14,15–20], learning and memory [15–21], fear and anxiety [5,15] or modulation of cortical excitability in humans [22]. However, a wide central distribution of TRPV1 has been questioned because TRPV1 reporter mice showed a restricted TRPV1 brain expression that it is conserved from rodents to humans [23]. We have very recently demonstrated that TRPV1 is highly concentrated in postsynaptic dendritic spines to asymmetric perforant path synapses in the dentate molecular layer [24] whose activation by anandamide triggers a type 1 cannabinoid (CB_1_) receptor-independent long-term depression (LTD) upon medial perforant path stimulation [19].

However, the knowledge of whether TRPV1 localizes at inhibitory synapses remains unresolved. The aim of this study was to investigate this by means of highly specific TRPV1 antibodies applied to the hippocampal dentate gyrus of TRPV1 wild-type (TRPV1-WT) and knock-out (TRPV1-KO) mice. The main results show a precise TRPV1 distribution mostly at postsynaptic dendrites receiving inhibitory synapses in the inner 1/3 of the molecular layer and in granule cell bodies of the dentate gyrus. These findings further support the anatomical substrate of TRPV1 in the brain.

## Materials and Methods

The protocols for animal care and use were approved by the appropriate Committee at the University of the Basque Country (CEBA/93/2010/GRANDESMORENO). Furthermore, the animal experimental procedures were carried out in accordance with The European Parliament and The Council of The European Union Directive of 22^nd^ of September 2010 (2010/63/UE) and Spanish regulations (R.D. 1201/2005; R.D. 53/2013). Great efforts were done in order to minimize the number and suffering of the animals used.

### Animal treatment

6 TRPV1-WT and 3 TRPV1-KO adult (3 month old) mice of either sex were used in this study. TRPV1-deficient mice (C57BL/6J background) [25] were originally obtained from The Jackson Laboratory (Strain Name: B6.129X1-*Trpv1* ^*tm1Jul*^/J, Bar Harbor, ME). TRPV1-KO construct information was fully described and shown by Caterina et al., [25]. Experimental animals were genotyped by polymerase chain reaction (PCR) under standard buffer conditions using the primer pair 5'-CCT GCT CAA CAT GCT CAT TG-3' and 5'-TCC TCA TGC ACT TCA GGA AA-3' for the wild-type locus. The primer pair 5'-CAC GAG ACT AGT GAG ACG TG-3' and 5'-TCC TCA TGC ACT TCA GGA AA-3' was used to detect a fragment in the Neo cassette, specific for the mutant TRPV1 locus. All four primers were used together in the reaction mix (94°C/3 min; 35x[94°C/30 sec, 64°C/1 min, 72°C/1 min]; 1x72°C/2 min; 1x10°C hold).

Homozygous TRPV1−/− and wild-type littermates (TRPV1+/+) from heterozygous breedings were used for experiments. They were deeply anesthetized by intraperitoneal injection of ketamine/xylazine (80/10 mg/kg body weight) and then transcardially perfused at room temperature (RT) with phosphate buffered saline (PBS 0.1M, pH 7.4) for 20 seconds, followed by the fixative solution made up of 4% formaldehyde (freshly depolymerized from paraformaldehyde), 0.2% picric acid and 0.1% glutaraldehyde in phosphate buffer (PB 0.1M, pH 7.4) for 10–15 minutes. Then, brains were removed from the skull and postfixed in the fixative solution for approximately one week at 4°C. Afterwards, brains were stored at 4°C in 1:10 diluted fixative solution until used.

### Preembedding immunogold method for TRPV1 electron microscopy (EM)

Coronal 50μm-thick hippocampal vibrosections were collected in 0.1M PB at RT. Then, they were preincubated in a blocking solution of 10% bovine serum albumin (BSA), 0.1% sodium azide and 0.02% saponine prepared in Tris-HCl buffered saline (TBS 1X, pH 7.4) for 30 minutes at RT. Sections were incubated with the primary goat anti-TRPV1 antibody (1:100, VR1 (P-19), affinity purified goat polyclonal antibody raised against a peptide mapping near the amino terminus of VR1 of rat origin, sc-12498, Santa Cruz Biotechnology) prepared in the blocking solution but with 0.004% saponin, for 2 days at 4°C. After several washes, tissue sections were incubated with 1.4 nm gold-labeled rabbit antibody to goat IgG (Fab´ fragment, 1:100, Nanoprobes Inc., Yaphank, NY, USA) prepared in the same solution as the primary antibody for 3 hours at RT. Tissue was washed overnight at 4°C and postfixed in 1% glutaraldehyde for 10 minutes. After several washes with double distilled water, gold particles were silver-intensified with a HQ Silver Kit (Nanoprobes Yaphank, NY, USA) for 12 minutes in the dark. Then, sections were osmicated, dehydrated and embedded in Epon resin 812. Finally, ultrathin sections were collected on mesh nickel grids, stained with lead citrate and examined in a PHILIPS EM208S electron microscope. Tissue preparations were photographed by using a digital camera coupled to the electron microscope. Figure compositions were made at 600 dots per inch (dpi). Labeling and minor adjustments in contrast and brightness were made using Adobe Photoshop (CS, Adobe Systems, San Jose, CA, USA).

Specificity of the immunostaining was assessed by incubation of the TRPV1 antiserum in TRPV1-KO hippocampal tissue in the same conditions as above.

### Statistical analysis of TRPV1 in the hippocampal dentate molecular layer

50-μm-thick hippocampal sections from TRPV1-WT (n = 6) and TRPV1-KO mice (n = 3) were cut at 80 nm. Electron micrographs (18,000–28,000X) were taken from grids (2 mm x 1 mm slot) with ultrathin sections obtained from areas at the same depth of the tissue block. Furthermore, to avoid false negatives, only ultrathin sections in the first 1.5 μm from the surface of the tissue block were examined. Positive labeling was considered if at least one immunoparticle was within approximately 30 nm from the plasmalemma. Metal particles on synaptic membranes were visualized and counted. The number of positive dendritic profiles was normalized to the total number of counted dendritic elements with symmetric synapses to identify the proportion of TRPV1 positive dendritic sections in TRPV1-WT versus TRPV1-KO. Density of TRPV1 immunolabeling was calculated on the same first 1.5 μm from the surface of tissue blocks with dendritic and granule somatic profiles. Image-J (version 1.43u NIH, USA) was used to measure the profiles´ area. Proportion of TRPV1 immunoparticles located at cytoplasms or dendritic membranes with symmetric synapses was obtained by normalizing the number of TRPV1 immunoparticles at these compartments versus total TRPV1 labeling on these postsynaptic profiles. Results were expressed as means of independent data points ± S.E.M. Statistical significance between groups was tested using unpaired Student’s t test (two-sided); *P*<0.05.

To establish the precise subcellular distribution of TRPV1 at inhibitory postsynaptic profiles relative to transmitter release sites, the number of immunoparticles was counted in 60-nm-wide segments starting at the edge of the symmetric postsynaptic membrane. The edge was defined as 0 with the synaptic or peri/extrasynaptic side to the left or right, respectively. As three samples analyzed did not differ in particle distribution (Kolmogorov-Smirnov test, *P*>0.19), the data were pooled. Image-J was used to measure gold particle’s distance counted from the edge of the symmetric membrane. Only profiles showing the whole membrane length from the synaptic edge (up to 360 nm) was used for counting.

All graphs and statistical analyses were performed using GraphPad software 5.0 (GraphPad Software Inc, San Diego, USA).

## Results

### TRPV1 immunolocalization at inhibitory synapses in the mouse dentate molecular layer

**Fig 1:**
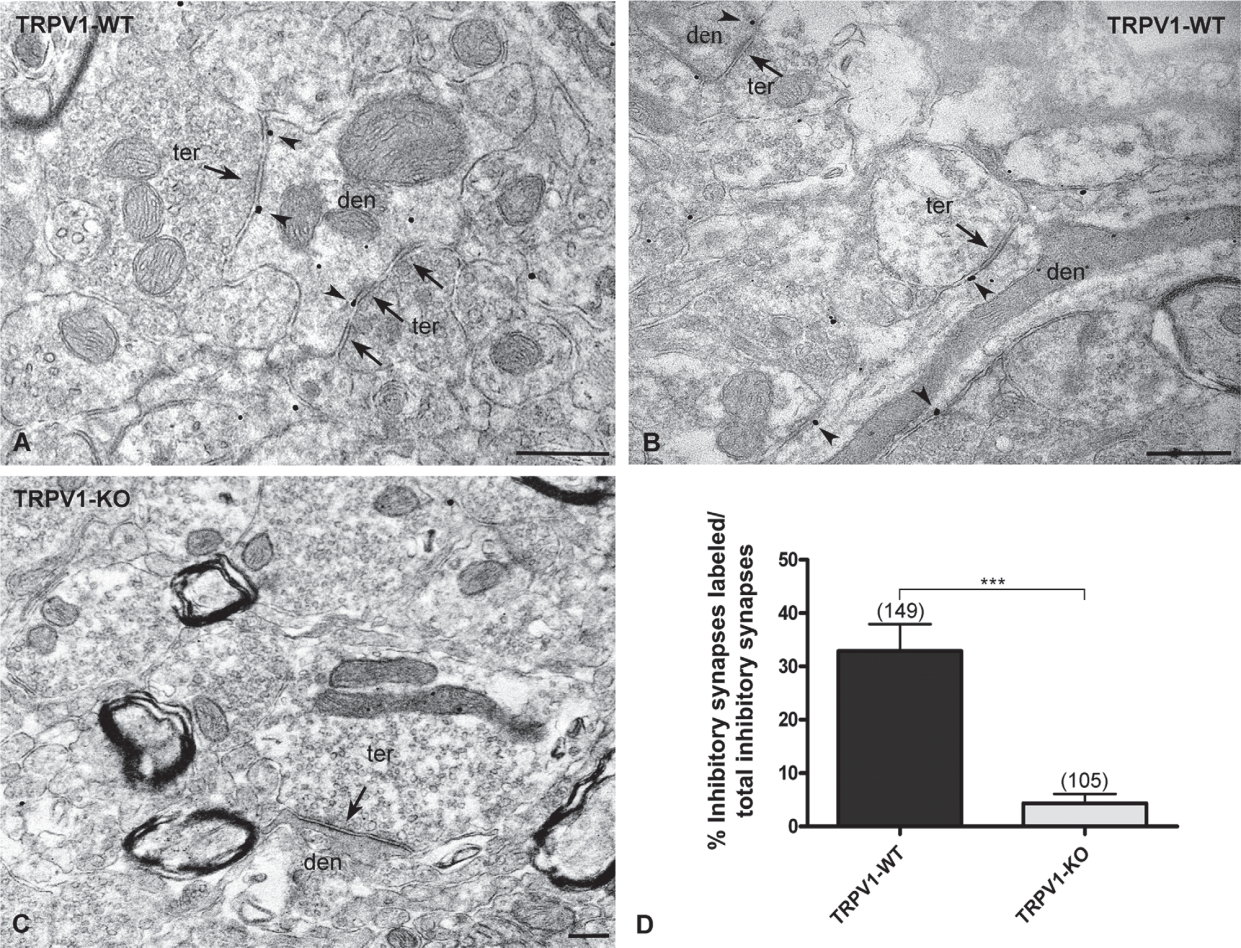
Immunolocalization of TRPV1 at symmetric synapses in mouse dentate ML. Preembedding immunogold method for electron microscopy (**A-C**). In TRPV1-WT (**A, B**), silver-intensified gold particles (arrowheads) are mostly distributed in dendritic (den) profiles but not in inhibitory synaptic terminals (ter). Importantly, TRPV1 immunolabeling is virtually abolished in the ML of TRPV1-KO mice (**C**). Scale bars: 0.5 μm. **D:** Percentages of TRPV1 positive dendritic profiles from the total analyzed dendritic sections with symmetric synapses (arrows in **A-C**) in the dentate ML of TRPV1-WT (dendrites: 32.89 ± 5.04%) and TRPV1-KO (dendrites: 4.76 ± 1.75%). Values in **D** mean ± SEM. ***P<0.*0001*.

**Fig 2:**
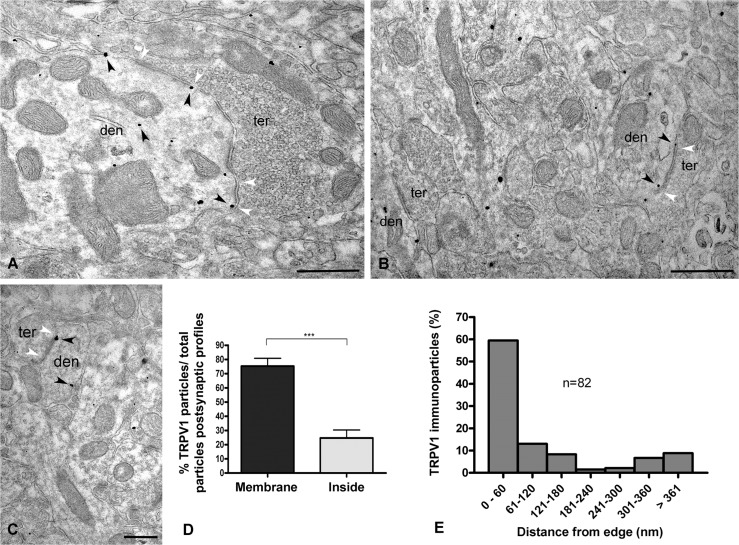
Subcellular distribution of TRPV1 at symmetric synapses in WT mouse dentate ML. Preembedding immunogold method for electron microscopy (**A-C**). TRPV1 immunoparticles are distributed in dendritic (den) sections receiving symmetric synapses (white arrowheads) from axon terminals (ter). Note that the metal particles (black arrowheads) are localized on the membranes and inside the dendritic profiles. Scale bars: 0.5 μm. **D:** Distribution of TRPV1 in dendrites (membrane: 75.25 ± 5.56%; inside: 24.75 ± 5.56%). Values mean ± SEM. (***P<0.*0001)*. **E:** Distribution of TRPV1 immunoparticles relative to the edge of postsynaptic membranes of symmetric synapses. The edge was defined as 0 with the peri/extrasynaptic side to the right. Note that the closest perisynaptic region (0–60 nm bin) contains the highest TRPV1 labeling (59.49%).

**Fig 3:**
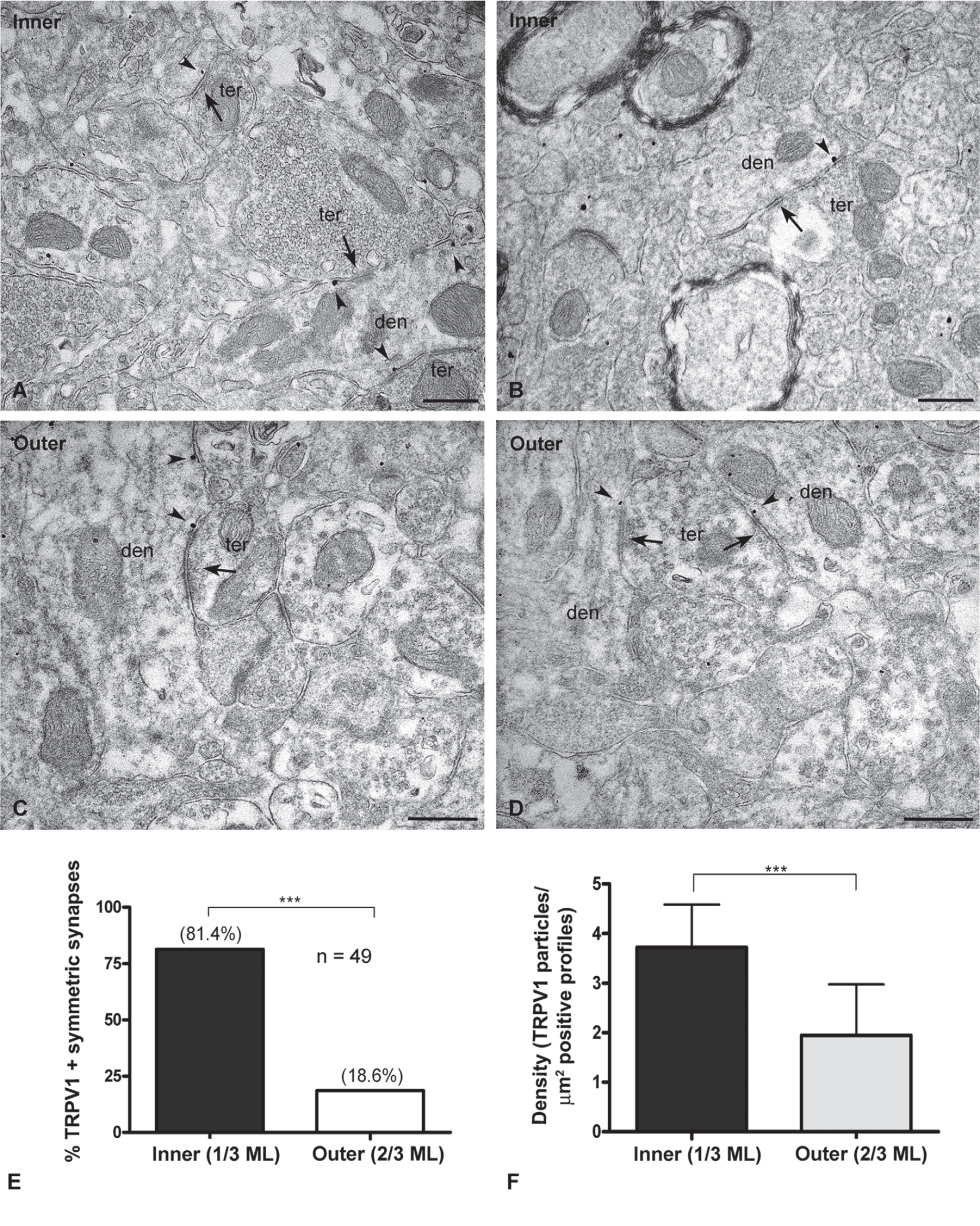
Immunolocalization of TRPV1 at dendrites in mouse dentate ML. Preembedding immunogold method for electron microscopy (**A-D**). TRPV1 immunolocalization (arrowheads) at dendritic (den) membranes receiving symmetric synapses (arrows) from axon terminals (ter) in the inner 1/3 (**A, B**) and outer 2/3 ML (**C, D**). Scale bars: 0.5 μm. **E:** Proportion of TRPV1 positive dendritic sections with symmetric synapses in the inner 1/3 (81.4%) and outer 2/3 (18.6%) of the dentate ML. **F:** Density of TRPV1 immunoparticles per area (part/μm^2^) in dendritic domains of the inner 1/3 (3.72 ± 0.85%) and outer 2/3 ML (1.95 ± 1.02%). Values mean ± SEM (***P<0.*0001*).

In the electron microscope, silver-intensified TRPV1 immunoparticles were revealed in thick postsynaptic dendrites receiving symmetric synapses in the dentate ML (Figs. 1A, B; 2A-C; 3A-D). About 33% of the total counted inhibitory synapses was TRPV1 immunopositive (Fig. 1D). Importantly, TRPV1 immunolabeling was residual in the TRPV1-KO hippocampal dentate ML (dendrites: 4.762% ± 1.76), meaning that the antibody used was highly specific (Fig. 1C, D). TRPV1-KO mice were confirmed by genotyping techniques.

So far, TRPV1-positive axon boutons with ultrastructural features of inhibitory synaptic terminals (pleomorphic vesicles and forming symmetric synapses) were not observed. Furthermore, TRPV1 immunolabeling at inhibitory synapses was more accumulated in membranes (∼75% of immunoparticles) than inside the postsynaptic dendrites (∼25%) (Fig. 2D). About 60% of the TRPV1 membrane immunoparticles were distributed at perisynaptic sites within 60 nm from the edge of the symmetric postsynaptic dendritic membrane, dropping to only 10% of the immunolabeling in the next bin between 60 and 120 nm (Fig. 2E).

**Fig 4:**
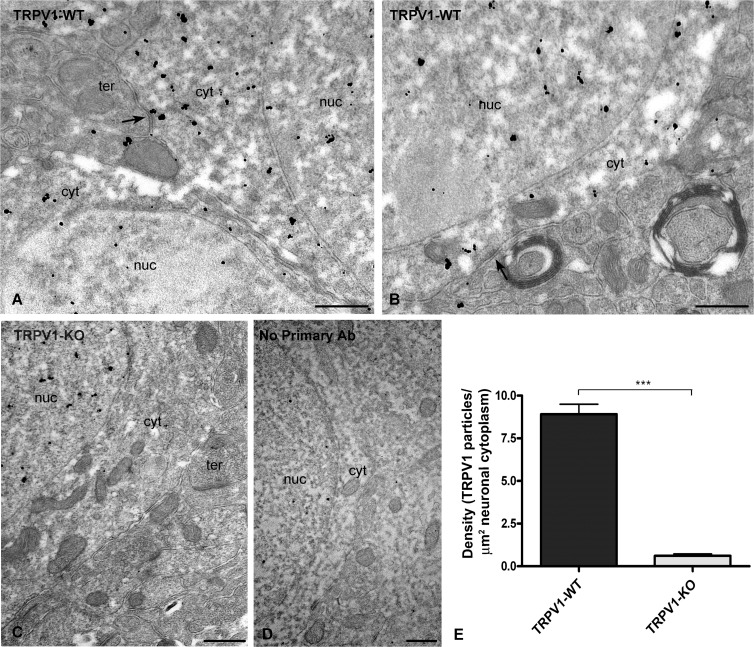
Immunolocalization of TRPV1 in granule cell perikarya of the mouse dentate ML. A high accumulation of TRPV1 immunoparticles is observed in the granule cytoplasm (cyt) of TRPV1-WT mouse (**A, B**). **A, B:** Arrows point to symmetric synapses made by axon terminals (ter) on the plasmalemma of granule cells. **C:** Notice the lack of TRPV1 labeling in the somatic cytoplasm of TRPV1-KO mice, but unspecific immunoparticles remain in the nucleus (nuc) that practically disappear after omission of the primary antibody (**D**). Scale bars: 0.5 μm. **E:** Density of TRPV1 immunoparticles per area (part/μm^2^) in the somatic cytoplasm of granule cells (TRPV1-WT 8.91 ± 0.58; TRPV1-KO 0.61 ± 0.11). Values mean ± SEM (***P<0.*0001*).

Of the 30% TRPV1 immunolabeled synapses, about 80% of them were distributed in the inner 1/3 and the rest (∼20%) in the outer 2/3 of the ML (Fig. 3E). Furthermore, the immunoparticle density in dendritic sections with symmetric synapses was higher in the molecular inner 1/3 (∼4 particles/μm^2^) than in the outer 2/3 of the layer (∼2 particles/μm^2^). This difference was statistically significant (Fig. 3F). Finally, TRPV1 was also localized in the granule cell bodies (Fig. 4). Although some scattered gold particles were on the plasmalemma at points were the somata received symmetric synapses, the bulk of metal particles was disperse in the cytoplasm (Fig. 4A, B). Again, TRPV1 perikaryal immunolabeling was almost null in the cytoplasm of granule cell bodies sections of TRPV1-KO mice, but it remained in the nucleus indicating that nuclear labeling was not specific (Fig. 4C), however, it almost disappeared after omission of the TRPV1 antibody (Fig. 4D). Finally, the density of cytoplasmic TRPV1 immunolabeling was relatively high (∼9 particles/μm^2^) (Fig. 4E).

## Discussion

In this study, we used a highly sensitive preembedding immunogold method for electron microscopy to elucidate whether TRPV1 is present at inhibitory synapses, in particular, in the dentate gyrus. Although the expression of TRPV1 in the hippocampus of reporter mice was confined to Cajal-Retzius cells [23], we have recently demonstrated that TRPV1 is localized at the excitatory perforant path synapses in the dentate ML [24]. Furthermore, functional findings support TRPV1 as the postsynaptic mediator of the anandamide-induced LTD at the excitatory medial perforant path synapses [19], in the nucleus accumbens [20] and the bed nucleus of the stria terminalis (BNST) [14]. There is a recent publication indicating that TRPV1 receptors/channels in dentate gyrus also regulate inhibitory synaptic transmission [26].

The subcellular and subsynaptic TRPV1 distribution pattern at inhibitory dentate ML synapses was similar to the described for the excitatory synapses in the BNST and the perforant path synapses [14,24]. Hence, silver-intensified gold particles have preferential perisynaptic localization. However, TRPV1 particle density at inhibitory synapses was lower than at the perforant path synapses [24]. Furthermore, there is a somehow complementary distribution pattern of TRPV1 in the dentate molecular layer because inhibitory synapses with thick TRPV1-immunopositive dendrites were more concentrated in the inner 1/3, whereas the TRPV1-immunopositive excitatory synapses were mostly in the outer 2/3, the termination zone of the perforant path [24].

About 25% of the TRPV1 immunoparticles were localized inside dendrites, as we reported already [24], and also a high accumulation of TRPV1 was in the cytoplasm of the granule cell bodies. Some previous studies indicated a neuronal cytoplasmic distribution of TRPV1 in several brain regions [11–13]. Although it is not clear the role of the intracellular TRPV1, it might regulate Ca^++^ release from intracellular stores needed for synaptic plasticity at excitatory synapses [19]. Intracellular TRPV1 in dendrites receiving inhibitory synapses and, in particular, the high amount of immunoparticles in the cytoplasm of granule cells may also serve functions for the control of inhibitory transmission [26].

The N-acyl phosphatidylethanolamine phospholipase D (NAPE-PLD), a biosynthetic enzyme of anandamide and its related bioactive compounds, is highly expressed in dentate granule cells [27], particularly in their excitatory mossy fiber boutons [28]. Furthermore, the NAPE-PLD-like immunoreactivity throughout the dentate molecular layer was reported to be more pronounced in the inner 1/3 [28]. Although the precise subcellular localization of NAPE-PLD relative to TRPV1 is presently unknown in dentate circuits, both proteins seem to co-exist in granule cells. Also, NAPE-PLD has been demonstrated in smooth endoplasmic reticulum of excitatory presynaptic terminals [28] and of postsynaptic dendrites and spines receiving excitatory synapses [14]. Hence, the generation of anandamide or other N-acyletanolamines (NAEs) by the calcium-dependent catalytic activity of NAPE-PLD may lead to the activation of TRPV1 in postsynaptic membranes and eventually triggering a long-term form of excitatory synaptic depression in the dentate gyrus [19] and BNST [14] or a depression of somatic inhibitory transmission in dentate granule cells [28].

The cannabinoid type 1 (CB_1_) receptors are highly localized in the glutamatergic mossy cell terminals in the inner 1/3 of the dentate molecular layer [29–32]. In contrast, TRPV1 is poorly expressed at postsynaptic excitatory synaptic membranes in the molecular innermost zone [24] but it is present at inhibitory synapses on dendrites as well as intracellularly in the granule cell bodies. Nevertheless, CB_1_ and TRPV1 seem to keep a close biological influence between each other because we have detected a significant increase of CB_1_ immunoreactivity in the inner 1/3 ML and an increase of DAGL-α and NAPE-PLD optical densities in dentate gyrus of TRPV1-KO versus WT (unpublished observations) which may have a functional benefit in certain pathological conditions, such as epilepsy.

As a conclusion, the TRPV1 localization in postsynaptic sites of inhibitory synapses and in granule cell bodies demonstrated here with specific antibodies and a highly sensitive immunocytochemical method provides the anatomical substrate for a TRPV1 regulation of inhibitory synaptic transmission in the dentate gyrus. Furthermore, the presence of TRPV1 at inhibitory and excitatory synapses in specific neuronal compartments of brain circuits recruited in physiological functions and also in disease prompts for a better understanding of the participation of TRPV1 in normal and abnormal brain.
